# RECQL4, Negatively Regulated by miR-10a-5p, Facilitates Cell Proliferation and Invasion via MAFB in Ovarian Cancer

**DOI:** 10.3389/fonc.2020.524128

**Published:** 2020-09-04

**Authors:** Li Guo, Yingwei Li, Chen Zhao, Jiali Peng, Kun Song, Long Chen, Ping Zhang, Hanlin Ma, Cunzhong Yuan, Shi Yan, Yan Fang, Beihua Kong

**Affiliations:** ^1^Department of Obstetrics and Gynecology, Qilu Hospital, Cheeloo College of Medicine, Shandong University, Jinan, China; ^2^Department of Obstetrics and Gynecology, Qingdao Municipal Hospital, Shandong University, Qingdao, China; ^3^School of Medicine, Cheeloo College of Medicine, Shandong University, Jinan, China

**Keywords:** RECQL4, MAFB, miR-10a-5p, proliferation, invasion, ovarian cancer

## Abstract

The high frequency of somatic copy number alterations, as opposed to point mutations, is considered a unique feature of ovarian cancer. Amplification-dependent overexpression of RecQ protein-like 4 (RECQL4), which participates in DNA replication and repair, mediates the development of various cancers, but its pathobiological and clinical roles are poorly understood. Here, using bioinformatics analysis, RECQL4 amplification was found to occur in 27% of ovarian cancer samples in the TCGA cohort. RECQL4 was found to be upregulated and associated with a poor prognosis based on the immunohistochemistry staining of ovarian cancer. Functionally, RECQL4 overexpression increased proliferation and invasion of ovarian cancer cells. RECQL4 silencing had the opposite effects. In addition, RECQL4 knockdown enhanced the sensitivity of ovarian cancer cells to cisplatin and PARP inhibitor (PARPi). Further mechanistic investigations revealed that MAFB was a downstream target of RECQL4. The oncogenic effect of RECQL4 was attenuated after MAFB knockdown. Moreover, RECQL4 overexpression was negatively regulated by the tumor suppressor miR-10a-5p. Collectively, these findings indicate that genomic amplification and low expression of miR-10a-5p contribute to RECQL4 overexpression in ovarian cancer. This is the first study to reveal the oncogenic functions and clinical significance of RECQL4 in ovarian cancer.

## Introduction

High-grade serous ovarian cancer (HGSOC) is the most common (70%) and aggressive subtype and is primarily responsible for the low survival rate of ovarian cancer (1). HGSOC was initially thought to arise from the ovarian surface epithelium, however, there is an increasing evidence suggesting that HGSOC originates in the fallopian tube (FT) (2). HGSOC is characterized by frequent TP53 mutations (96%), somatic copy number alterations, and deregulated NOTCH and FOXM1 signaling (3). Recent studies suggested that the majority of genetic changes were not somatic point mutations but rather somatic copy-number alterations (SCNAs) (4). SCNAs were found to be much more prevalent (113/489) than point mutations in integrated genomic analysis of HGSOC. The most common driver genes, such as MYC, CCNE1, and MECOM, were amplified in more than 20% of HGSOC cases (3). MYC amplification maintains the oncogenic growth of HGSOC cells, and it can be targeted therapeutically (5). Cyclin E1 is overexpressed in up to 50% of HGSOC cases, and CCNE1 silencing reduces ovarian cancer cell viability (6).

RecQ protein-like 4 (RECQL4) helicase is a molecular motor that unwinds DNA, a process essential during DNA replication and repair (7). RECQL4 regulates DNA replication (8), telomere maintenance (9) and genome stability (10–12). RECQL4 promotes both nonhomologous end joining and homologous recombination (13, 14). RECQL4 deficiency in Rothmund–Thomson syndrome leads to an increased incidence of osteosarcoma or lymphoma (15). In contrast, elevated expression of RECQL4 is observed in prostate cancer, breast cancer, and hepatocellular carcinoma (16–18). However, the expression and biological roles of RECQL4 in ovarian cancer remain undefined. Here, we studied the expression, biological functions, and clinical significance of RECQL4 in ovarian cancer, and the reasons for high expression of RECQL4 were further analyzed in this study.

## Materials and Methods

### Tissue Samples

Ovarian cancer samples were obtained from surgical resections. According to the latest view that majority of serous ovarian cancer originates from the fimbria (19), the FT is selected as the control. FT tissues were extracted from the benign tumors of patients undergoing hysterectomy and adnexectomy. In total, 157 ovarian cancer and 54 normal FT tissues were obtained for tissue microarrays (TMAs) analysis. The 40 fresh-frozen ovarian cancer tissues and 20 normal FTs were used for western blot and qRT-PCR analysis. All patients were provided with the informed consent. The ovarian cancer tissue samples of fresh-frozen tissues and TMA sections are HGSOC.

### Bioinformatics Analysis

TCGA ovarian cancer (TCGA, Nature 2011) data were downloaded from online website cbioportal^1^. The original data of TCGA PanCancer is from Pan-Cancer Atlas^2^. The relative expression of RECQL4 in TCGA pan-cancer was from UALCAN^3^. The data of gene expressions and genomic amplification in ovarian cancer cells was obtained from CCLE^4^. The ovarian cancer tissue samples from TCGA and UALCAN are HGSOC. The ovarian cancer cell lines from CCLE and tissue samples from Kaplan–Meier Plotter are not limited to HGSOC.

### Immunohistochemistry

We performed hematoxylin-eosin (HE) staining to determine the location of the ovarian cancer lesion. The ovarian cancer TMAs were constructed by obtaining two 1-mm diameter cores from each tumor at two different neighboring sites with a semiautomatic tissue arrayer. Every tumor consisted of two cores to ensure accuracy of final analysis to further eliminate the effect of tumor heterogeneity as far as possible.

The slides were deparaffinized and rehydrated, and antigen retrieval was performed with citric acid treatment. Upon blocking endogenous peroxidase activity, nonspecific protein–protein interactions were blocked by treatment with goat serum. Sections were incubated with primary antibodies overnight at 4°C and then were labeled with the indicated streptavidin-peroxidase secondary antibodies. The sections were stained with a DAB detection system and haematoxylin. The sections were dehydrated in a gradient ethanol series, and two trained pathologists scored each sample according to the extent and intensity of staining. The intensity of staining was scored as 0 (negative), 1 (weak), 2 (moderate), or 3 (strong). The extent of staining was based on the percentage of positive tumor cells: 1 (0–25%), 2 (26–50%), 3 (51–75%), and 4 (76–100%). The final immunohistochemistry (IHC) score staining was generated by multiplying the percentage score with the staining intensity score. Each sample has two duplicates and the final score was the average score of the duplicates. Each case was considered to have low RECQL4 expression if the final score was less than 4 and high RECQL4 expression if the final score was 5 or greater. If the intensity of staining is strong (scored as 3), and the extent of staining is 100% (scored as 4), the total score of the staining is 12 (3 times 4 equals 12). The ovarian cancer tissue samples of TMA sections are HGSOC.

### RNA Isolation and qRT-PCR

Tissues were lysed for total RNA extraction using TRIzol, and RNA was reverse transcribed to generate cDNA using PrimeScript RT Kits (Takara). qRT-PCR was performed using SYBR-Green qPCR master mixes. ACTB was used as an internal control. The ovarian cancer tissue samples used in the qRT-PCR assay are HGSOC. The primers used in this study are listed in Supplementary Table S1.

### Western Blotting

Tissues were lysed in RIPA supplemented with PMSF and NaF. Lysates (30 μg) were resolved on SDS-PAGE gels, and proteins were transferred to PVDF membranes, which were then blocked in 5% milk for 1 h. Membranes were incubated with primary antibodies at 4°C overnight and were subsequently labeled with the corresponding HRP-conjugated secondary antibodies for 2 h. Protein bands were detected using ECL and were quantitated using ImageJ 1.47. β-actin was used as an endogenous control. The antibodies used in this study were from an Epithelial-Mesenchymal Transition Kit, Cell Cycle Regulation Antibody Sampler Kit, and Apoptosis Antibody Sampler Kit. The RECQL4 antibody was purchased from Novus Biologicals. β-actin antibody was obtained from Sigma-Aldrich. MAFB antibody was purchased from Sangon Biotech. The ovarian cancer tissue samples used in the western blotting assay are HGSOC.

### Cell Lines and Cell Culture

A2780, A2780/DDP, SKOV3, and UWB1.289 cells were grown in RPMI 1640 plus 10% FBS. HEY and HEK293T cells were grown in DMEM plus 10% FBS. All cells were grown in standard cell culture conditions (37°C, 5% CO_2_). The lines were confirmed using unique short tandem repeat (STR) analyses.

### Cell Transfection and the Production of Stable Cell Lines

The RECQL4 overexpression vector (GV492-RECQL4) and control vector (GV492) were purchased from GeneChem. The vectors carrying shRNAs targeting RECQL4 were purchased from Sigma-Aldrich, United States. Lentiviral vectors were packaged in HEK293T cells using psPAX2 and pMD2.G to produce lentivirus particles. The supernatant was collected 24 and 48 h post-transfection and filtered through 0.45 μm PVDF membranes. Ovarian cancer cells were infected with the lentivirus particles for 24 h and selected for 7 days in culture medium containing 2 μg/ml puromycin.

siRNAs targeting RECQL4 or MAFB and mimics and inhibitors of miR-10a-5p were obtained from GenePharma. The sequences of siRNAs are shown in Supplementary Table S1.

### Cell Viability Assays

Cells (1 × 10^3^) were seeded into 96-well plates for 1–6 days, and CCK-8 solution was added to assess cell proliferation. The absorbance at 450 nm was measured using a microplate reader.

### Colony Formation Assay

Ovarian cancer cells (800–1000 cells) were grown in 6-well plates for 10–14 days. The colonies were fixed using methanol and stained using crystal violet. Colonies (≥50 cells/colony) were counted under a microscope.

### Cell Migration and Invasion Assays

Ovarian cancer cells were seeded into 24-well chambers with or without a coat of Matrigel. Cells (1 × 10^5^ per well) were seeded into the upper chamber in serum-free medium, and medium containing 20% FBS was added to the lower chamber. The cells that penetrated through the membrane were assessed with methanol fixation and 0.1% crystal violet staining.

A wound healing assay was used to evaluate cell motility in ovarian cancer cells after RECQL4 overexpression or knockdown. Ovarian cancer cells were grown to confluency in 6-well plates (2 × 10^5^ per well), and a 20 μl pipette tip was used to produce a scratch across the cell monolayer. Serum-free medium was added to the well, and the distances between the scratches were measured at the indicated times.

### Cell Cycle and Apoptosis Assessments

Ovarian cancer cells were transfected with an RECQL4 siRNA and control. Approximately 72 h later, cells were collected and stained with PI for cell cycle analysis. The cells were stained with annexin V-FITC and PI for apoptosis assessments. Cell cycle and apoptosis was evaluated by flow cytometry.

### High-Throughput Differential Gene Expression Analysis

The high-throughput RNA-seq experiments were conducted by Berry Genomics (Beijing, China). mRNA-seq library was prepared for sequencing following standard Illumina protocols. In brief, total RNAs from si-RECQL4 and control cells were extracted using TRIzol reagent and treated with RNase-free DNase I to remove genomic DNA. mRNA was extracted with Dynabeads Oligo(dT). Double-stranded complementary DNAs are synthesized using Superscript II reverse transcriptase and random hexamer primers. The cDNAs were then fragmented by nebulization and the standard Illumina protocol was followed thereafter, to create the mRNA-seq library. For the data analysis, basecalls were performed using CASAVA. Reads were aligned to the genome using the split read aligner TopHat (v2.0.7) and Bowtie 2, using default parameters. The RNAseq data had been uploaded to GEO, and the accession number is GSE153839.

### Luciferase Assays

The 3′UTR sequences of RECQL4 were subcloned into pmirGLO (Promega) to produce wild-type and mutant luciferase vectors. The 3′UTR construct and mimics or scrambled control of miR-10a-5p were cotransfected into HEK293T cells using Lipofectamine 2000. Luciferase activity was measured 24 h post-transfection, and the relative luciferase activity was determined by calculating the ratio of the firefly luciferase and Renilla luciferase values.

### Nude Mouse Xenograft Models

Nude female BALB/c mice aged 4–5 weeks were obtained from NBRI of Nanjing University (Nanjing, China). After acclimatization for 1 week, mice were divided into two groups (*n* = 5). For the tumorigenesis assay, 5 × 10^6^ cells overexpressing RECQL4 and the control were subcutaneously injected into the axilla. Mice were sacrificed 2 weeks post-injection, and tumor masses were assessed.

### Statistical Analysis

Differential expression was analyzed using GraphPad Prism 5.0. Groups were compared for significant differences using Student’s *t*-test or Chi-square test. Survival analysis was performed using Kaplan–Meier and log-rank tests. ^∗^*P* < 0.05 and ^∗∗^*P* < 0.01 were considered statistically significant.

## Results

### Upregulated RECQL4 Predicts Poor Prognosis in Ovarian Cancer Cases

**FIGURE 1:**
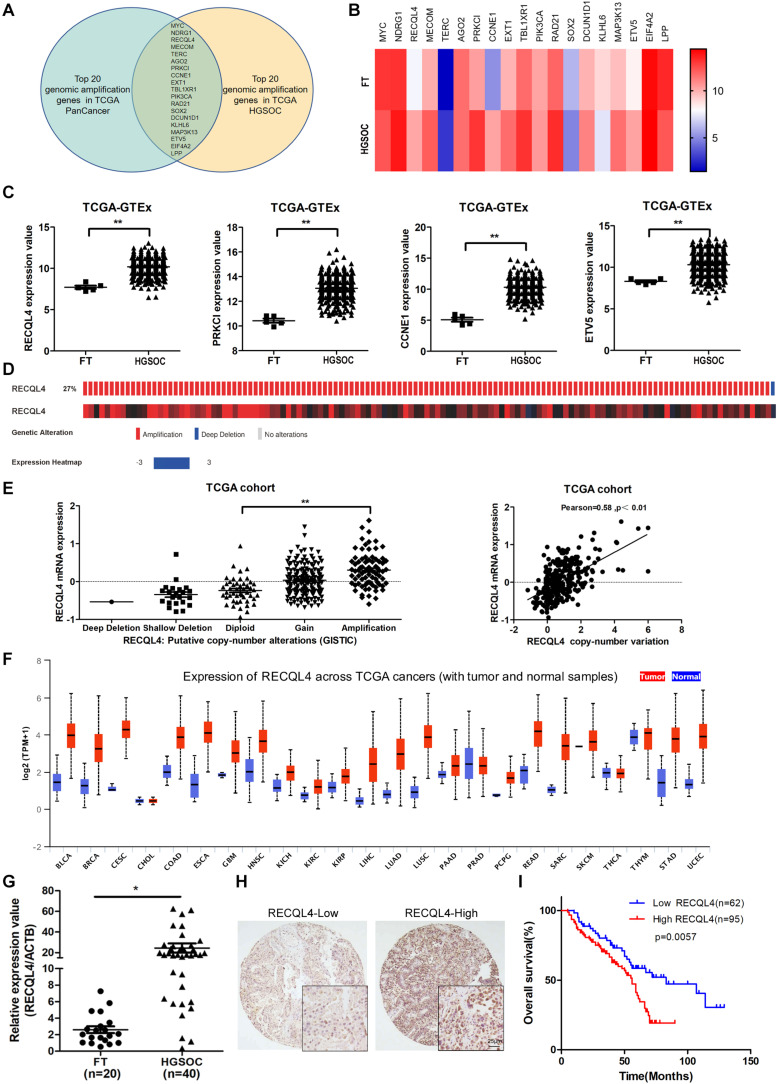
RECQL4 is overexpressed in ovarian cancer. **(A)** Venn diagram of 19 hub genes between the top 20 genomic amplified genes in ovarian cancer and pan-cancer from TCGA. **(B)** Heatmap of the top 20 genes expressed between ovarian cancer and normal fallopian tube tissues using the data from TCGA-GTEX. **(C)** Relative mRNA expression of RECQL4, PRKCL, CCNE1, and ETV5 in ovarian cancer and normal fallopian tube tissues using the data from TCGA-GTEX. **(D)** Genetic alterations of RECQL4 in ovarian cancer in the cohort from TCGA. **(E)** Relative mRNA expression of RECQL4 from samples with different copy number variation statuses. Correlation analysis between RECQL4 amplification and mRNA expression in TCGA ovarian cancer samples. **(F)** Analysis of differential expression of RECQL4 across TCGA pan-cancers using UALCAN **(G)** qRT-PCR analysis of RECQL4 mRNA expression between 40 ovarian cancer and 20 fresh-frozen fallopian tube tissues. **(H)** Representative IHC of RECQL4 in the ovarian cancer samples based on our tissue microarrays. **(I)** Kaplan–Meier analysis of the effect of RECQL4 protein expression on the overall survival of ovarian cancer patients based on follow-up information from our tissue microarrays. The ovarian cancer tissue samples from TCGA and UALCAN are HGSOC. The ovarian cancer cell lines from CCLE are not limited to HGSOC. The ovarian cancer tissue samples used in the qRT-PCR assay and tissue microarray sections are HGSOC. The list of pan-cancer abbreviations were described as follows. BLCA, bladder urothelial carcinoma; BRCA, breast invasive carcinoma; CESC, cervical squamous cell carcinoma and endocervical adenocarcinoma; CHOL, cholangiocarcinoma; COAD, colon adenocarcinoma; ESCA, esophageal carcinoma; GBM, glioblastoma multiforme; HNSC, Head and Neck squamous cell carcinoma; KICH, kidney chromophobe; KIRC, kidney renal clear cell carcinoma; KIRP, kidney renal papillary cell carcinoma; LIHC, liver hepatocellular carcinoma; LUAD, lung adenocarcinoma; LUSC, lung squamous cell carcinoma; PAAD, pancreatic adenocarcinoma; PRAD, prostate adenocarcinoma; PCPG, pheochromocytoma and paraganglioma; READ, rectum adenocarcinoma; SARC, sarcoma; SKCM, skin cutaneous melanoma; THCA, thyroid carcinoma; THYM, thymoma; STAD, stomach adenocarcinoma; UCEC, uterine corpus endometrial carcinoma. *P < 0.05, **P < 0.01.

Bioinformatics analysis of genomic copy number variations (CNVs) of ovarian cancer and pan-cancer in the TCGA cohort showed that CNVs was ubiquitous in tumors (Supplementary Figure S1A). Moreover, the top 20 genomic amplifications in ovarian cancer and pan-cancer were almost identical (19/20) (Figure 1A and Supplementary Figure S1B). Using data from TCGA and GTEX, the mRNA expression levels of RECQL4, PRKCL, CCNE1, and ETV5 were found to be increased in ovarian cancer compared to normal FT tissues (Figures 1B,C). RECQL4 expression was found to be higher in ovarian cancer tissues than in normal tissues in the GSE12470 and GSE26712 datasets (Supplementary Figure S1C). Moreover, RECQL4 was amplified in approximately 27% of ovarian cancer cases (Figure 1D), and its amplification was correlated with high mRNA levels (Figure 1E). High levels of RECQL4 mRNA expression were also found to be positively related with its CNV in the ovarian cancer cell line using data from the CCLE dataset (Supplementary Figures S1D,E). qRT-PCR was applied to assess the relative expression of RECQL4 between ovarian cancer and FT tissues. As shown in Figure 1G, RECQL4 mRNA expression was significantly upregulated in ovarian cancer. In addition, RECQL4 protein expression levels in ovarian cancer cells was markedly higher than it was in the normal FT cell line FTE187 (Supplementary Figure S1F). Furthermore, RECQL4 mRNA expression was found to be significantly overexpressed across different human tumor types (Figure 1F).

To assess the correlation between RECQL4 and ovarian cancer patient characteristics, IHC staining analysis was used to measure RECQL4 protein expression in 157 ovarian cancer and 54 normal FT tissues. Positive RECQL4 staining was concentrated in the nucleus (Figure 1H). A total of 60.51% of patients had high RECQL4 expression (95/157), and the proportion in the RECQL4 low expression group was 39.49%. Kaplan–Meier analysis revealed that ovarian cancer patients with high RECQL4 expression had reduced overall survival (OS) (Figure 1I). High mRNA level of RECQL4 indicated poor OS and progression-free survival (PFS), according to the data from the online analysis website Kaplan–Meier Plotter^5^ (Supplementary Figures S1G,H). We next assessed the correlation between RECQL4 protein expression and clinicopathological parameters based on IHC. Elevated RECQL4 expression positively correlated with CA125 levels and omental metastasis (Table 1).

**TABLE 1:** Correlation analysis between RECQL4 expression and clinicopathologic characteristics of ovarian cancer patients based on immunohistochemistry staining.

Characteristics	Number of cases	RECQL4 expressionh		*P*-value
		Low	High	
Age (years)				0.116
<50	49	14	35	
≥50	108	45	63	
CA125(U/ml)				0.009
<600	67	33	34	
≥600	90	26	64	
FIGO stage				0.416
I + II	37	16	21	
III +IV	120	43	77	
Lymph node metastasis				0.692
Negative	64	26	38	
Positive	22	10	12	
Omentum metastasis				0.010
Negative	53	28	25	
Positive	87	27	60	
Cisplatin status				0.041
Sensitive	26	13	13	
Resistant	23	5	18	

These findings indicated that RECQL4 is overexpressed in ovarian cancer and correlates with a poor prognosis of ovarian cancer patients.

### RECQL4 Enhances the Proliferation Ability of Ovarian Cancer Cells

**FIGURE 2:**
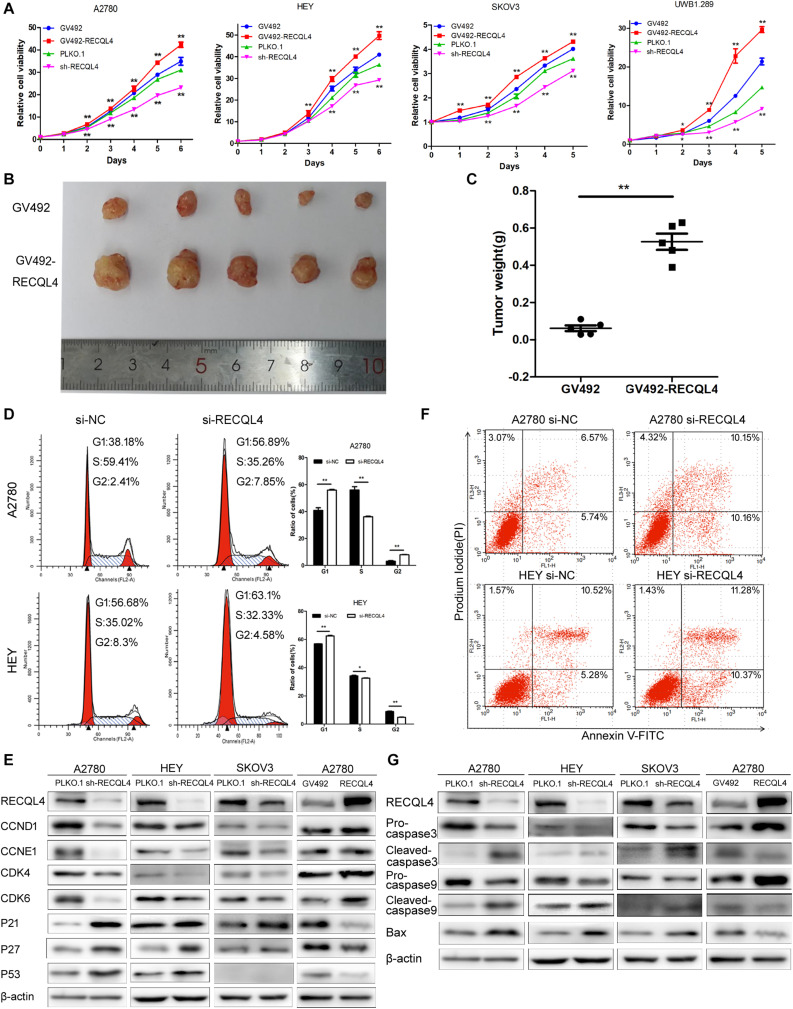
RECQL4 promotes cell growth *in vitro* and *in vivo*, while silencing it increases apoptosis. **(A)** Growth curves were generated to evaluate the effect of RECQL4 knockdown and overexpression on ovarian cancer cells. **(B)** Image of tumors isolated from nude mice with tumor xenografts derived from the indicated groups. **(C)** The xenografts were weighed and compared. **(D)** Cell cycle assessments of ovarian cancer cells transfected with si-RECQL4 and si-NC. **(E)** Western blot analysis of cell cycle-related proteins after RECQL4 knockdown and overexpression. **(F)** A2780 and HEY cells were transfected with si-RECQL4 and si-NC and were treated with 4 μg/ml cisplatin for 24 h to induce apoptosis. Representative annexin V/PI staining of treated ovarian cancer cells. **(G)** Western blot analysis of apoptosis-related proteins after RECQL4 knockdown and overexpression. Data are the mean ± SEM of at least three independent experiments. *P < 0.05, **P < 0.01.

To investigate the biological functions of RECQL4, we established RECQL4 overexpression and knockdown cell lines. RECQL4 overexpression enhanced the viability of cells and their colony-forming ability. RECQL4 silencing inhibited cell growth and reduced the number of cell clones (Figure 2A and Supplementary Figure S2A). The effects of RECQL4 during tumorigenesis of ovarian cancer were explored *in vivo*. HEY cells overexpressing GV492-RECQL4 and the control cells were subcutaneously injected into nude mice, and the results showed that the volume and weight of tumors derived from RECQL4-overexpressing cells were increased compared to those of tumors derived from control cells (Figures 2B,C).

Flow cytometry was used to further assess the effects of RECQL4 on cell cycle progression. Compared to control cells, A2780 and HEY cells with RECQL4 knocked down exhibited increased G1 arrest and decreased S-phase progression (Figure 2D). RECQL4 knockdown decreased the expression of CCND1, CCNE1, CDK4, and CDK6 and increased P21, P27, and P53 expression, and the opposite results were observed in RECQL4-overexpressing cells through western blot assay (Figure 2E and Supplementary Figure S2E). The effect of RECQL4 knockdown on the apoptosis of ovarian cancer cells was evaluated by Flow cytometry. The results showed that the proportion of apoptotic cells increased upon RECQL4 silencing (Figure 2F and Supplementary Figures S2C,D). Similarly, as shown in Figure 2G and Supplementary Figure S2F, RECQL4 silencing increased the expression of the following apoptosis-related proteins: cleaved Caspase 9, cleaved Caspase 3, and Bax.

These data collectively suggest that RECQL4 overexpression promotes the proliferation of ovarian cancer cells and its silencing promotes G1/S phase arrest and facilitate apoptosis.

### RECQL4 Facilitates the Invasion Ability of Ovarian Cancer

**FIGURE 3:**
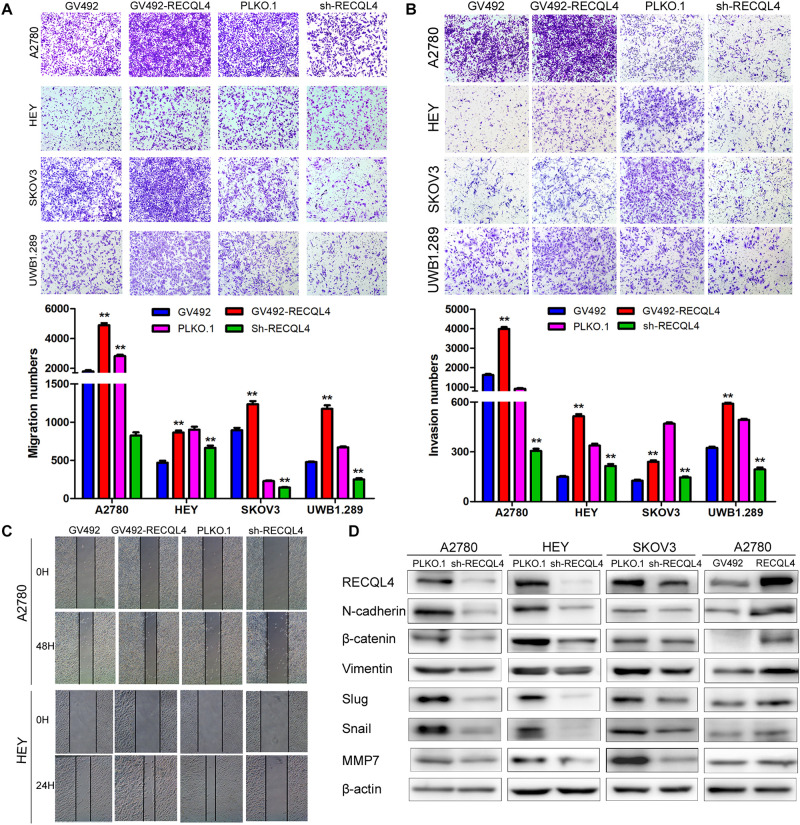
RECQL4 promotes the invasion of ovarian cancer cells. **(A)** Transwell assays were used to evaluate the migration ability of ovarian cancer cells after RECQL4 knockdown or overexpression. **(B)** Transwell assays were performed to evaluate the invasion of ovarian cancer cells after RECQL4 knockdown or overexpression. **(C)** Wound healing assays were applied to evaluate the motility of ovarian cancer cells after RECQL4 knockdown or overexpression. **(D)** Western blot analysis of EMT-related markers after RECQL4 knockdown and overexpression. Data are the means ± SEM of at least three independent experiments. **P < 0.01.

A transwell assay was then conducted to investigate the effect of RECQL4 on ovarian cancer cell invasion. RECQL4 overexpression in A2780, HEY, SKOV3, and UWB1.289 cells increased the migratory and invasive abilities. Correspondingly, RECQL4 knockdown decreased ovarian cancer cell migration and invasion (Figures 3A–C and Supplementary Figures S3A,B). Western blot analysis of epithelial-mesenchymal transition (EMT) markers revealed that RECQL4 silencing decreased the expressions of N-cadherin, β-catenin, vimentin, slug, snail, and MMP7. Moreover, RECQL4 overexpression increased the expressions of these mesenchymal markers (Figure 3D and Supplementary Figure S3C). Accordingly, these results indicated that RECQL4 overexpression increases the invasion of ovarian cancer cells.

### RECQL4 Silencing Increases the Sensitivity of Ovarian Cancer Cells to Olaparib and Cisplatin

Correlation analysis of RECQL4 protein expression and clinical characteristics, based on IHC staining, showed that high expression of RECQL4 was associated with cisplatin resistance status (Table 1). Ovarian cancer patients treated with cisplatin with high RECQL4 expression had shorter OS than those with low RECQL4 expression according to the online analysis website Kaplan–Meier Plotter (Supplementary Figure S1I). RECQL4 expression was also significantly higher in cisplatin-resistant A2780/DDP cells than in A2780 cells (Supplementary Figure S4A), suggesting a role for RECQL4 in the cisplatin resistance of ovarian cancer. Ovarian cancer cells were treated with different concentrations of cisplatin for 48 h, and RECQL4 protein expression increased in a dose-dependent manner (Supplementary Figure S4B). CCK-8 assays showed that the IC50 of cisplatin in ovarian cancer cells declined after RECQL4 knockdown (Supplementary Figure S4C). Clonogenic assays also revealed a reduced number of viable cell colonies in RECQL4-knockdown cells following cisplatin treatment for 48 h (A2780/DDP for 72 h) (Supplementary Figures S4D,E).

**FIGURE 4:**
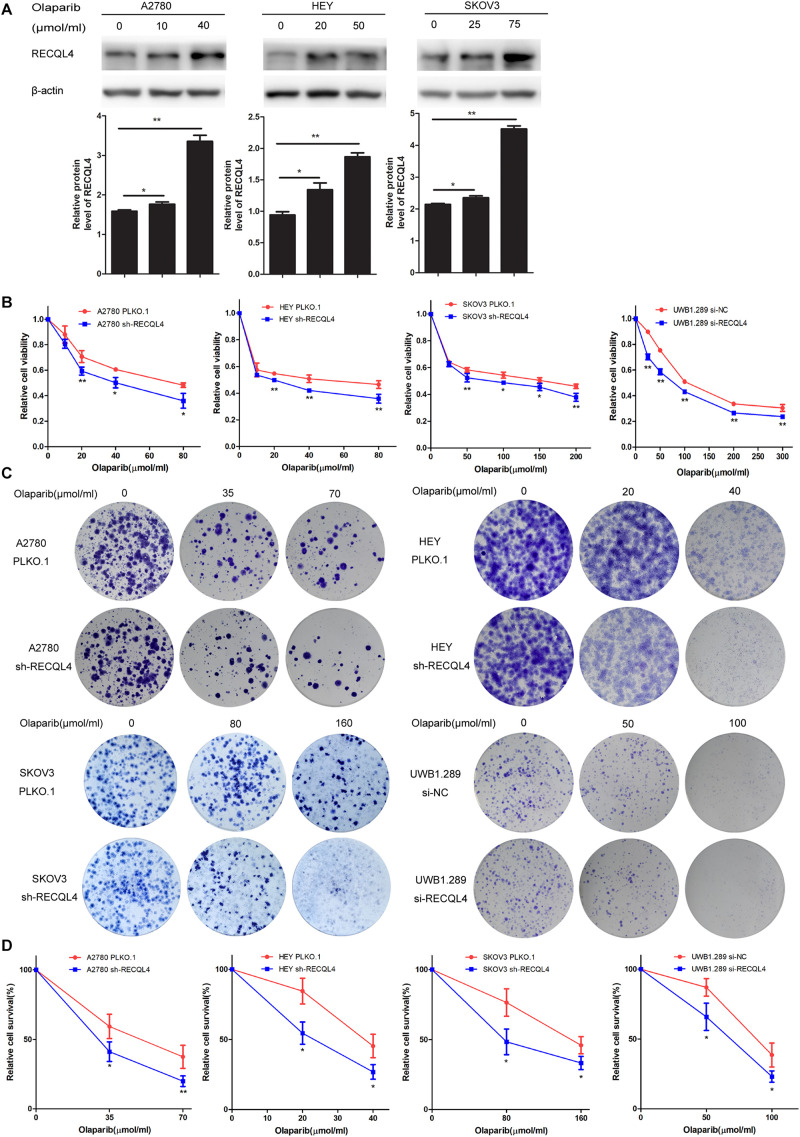
Knockdown of RECQL4 increased the sensitivity of ovarian cancer cells to olaparib treatment *in vitro*. **(A)** Relative protein expression of RECQL4 was measured after treatment with graded concentrations of olaparib for 4 days. **(B)** Growth curves were generated to evaluate the effect of RECQL4 knockdown on the viability of ovarian cancer cells treated with graded concentrations of olaparib for 4 days. **(C)** A clonogenic assay was performed to assess the colony formation ability of RECQL4 knockdown on the number of clones from ovarian cancer cells treated with increasing concentrations of olaparib for 4 days. **(D)** An IC50 graph summarizing the data shown in panel **(C)**. Values are the mean ± SEM from three independent experiments. *P < 0.05, **P < 0.01.

We further evaluated the role of RECQL4 in enhancing the sensitivity of ovarian cancer cells to olaparib. A2780, HEY, SKOV3, and UWB1.289 cells were exposed to increasing concentrations of olaparib for 4 days. RECQL4 levels were also found to increase in a dose-dependent manner (Figure 4A). Both CCK-8 assays and clonogenic assays confirmed that RECQL4 silencing enhanced the sensitivity to olaparib (Figures 4B–D). Taken together, these data show that ovarian cancer cells with RECQL4 knocked down are more sensitive to cisplatin and olaparib treatment than control cells.

### MAFB Is an Important Downstream Effector of RECQL4, and Its Knockdown Suppresses Ovarian Cancer Proliferation and Invasion Phenotypes

**FIGURE 5:**
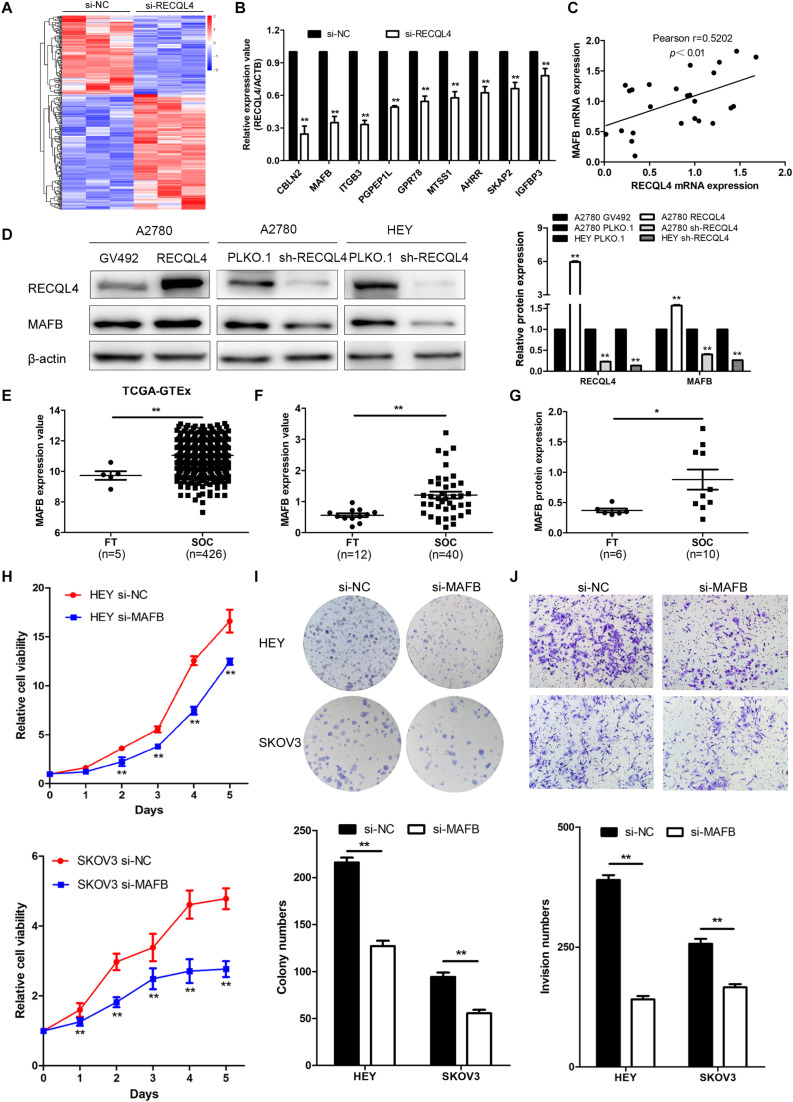
MAFB is an important downstream effector of RECQL4, and its knockdown suppresses ovarian cancer proliferation and invasion phenotypes. **(A)** Heatmap of differentially expressed gene profiles in HEY cells transfected with si-RECQL4 and si-NC. **(B)** qRT-PCR was used to validate representative differentially expressed genes. **(C)** Correlation analysis between RECQL4 and MAFB expression in ovarian cancer fresh-frozen tissues. **(D)** Western blot analysis of MAFB protein expression after RECQL4 knockdown and overexpression. **(E)** Relative mRNA expression of MAFB was analyzed between ovarian cancer and fallopian tube tissue samples in the cohort from TCGA-GTEX. **(F)** qRT-PCR analysis of MAFB relative mRNA expression in 12 fallopian tube and 40 ovarian cancer tissues. **(G)** Western blot analysis of MAFB protein expression levels in 6 fallopian tubes and 10 ovarian cancer tissues. **(H)** A growth curve was generated to evaluate the effect of MAFB on the proliferation of ovarian cancer cells. **(I)** Colony formation assays were used to assess the effect of MAFB on the proliferation of ovarian cancer cells. **(J)** Transwell assays were applied to evaluate the effect of MAFB on the invasion of ovarian cancer cells. The ovarian cancer tissue samples used in the qRT-PCR and western blot assay are HGSOC. *P < 0.05, **P < 0.01.

To explore the mechanism by which RECQL4 drives ovarian cancer malignancy, we performed RNA-seq in HEY cells with RECQL4 knockdown compared to control cells. A total of 302 differentially expressed genes (DEGs) were identified (fold change ≥2, *P* < 0.05); there were 179 upregulated and 123 downregulated genes (Figure 5A). qRT-PCR was performed to validate these DEGs (Figure 5B). As shown in Figure 5C, MAFB mRNA expression positively correlated with RECQL4 expression in ovarian cancer tissues. MAFB protein expression was found to be increased after RECQL4 overexpression but decreased after RECQL4 knockdown (Figure 5D). Moreover, MAFB was upregulated in ovarian cancer tissues compared to FTs in the cohort from TCGA-GTEX (Figure 5E). MAFB mRNA and protein were found to be highly expressed in fresh-frozen SOC and FT tissues, as assessed by qRT-PCR and western blot assays (Figures 5F,G).

To assess the biological function of MAFB in ovarian cancer cells, MAFB was silenced in HEY and SKOV3 cell lines. MAFB knockdown inhibited cell viability and colony-forming ability (Figure 5H,I). Transwell assays revealed that MAFB knockdown decreased the invasion ability of ovarian cancer cells (Figure 5J). These data imply that MAFB is targeted by RECQL4 and its knockdown inhibited proliferation and invasion of ovarian cancer cells.

### The Oncogenic Effects of RECQL4 Are Alleviated by MAFB Silencing

**FIGURE 6:**
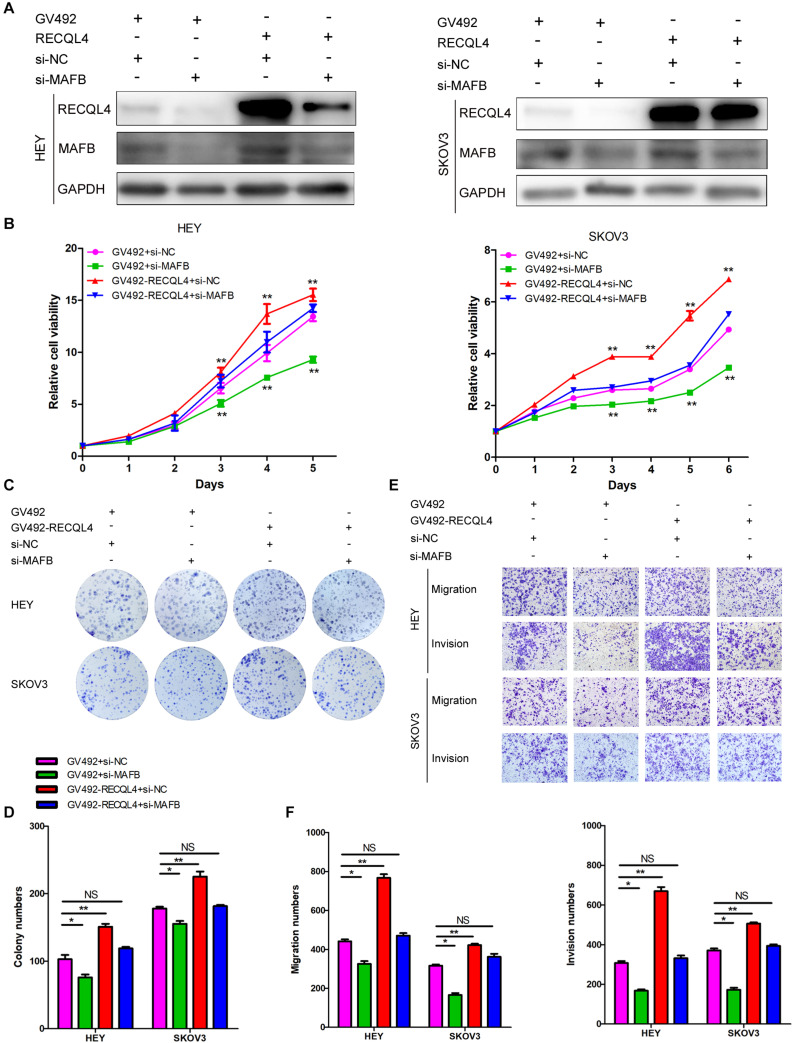
RECQL4 promotes cell proliferation and mobility by upregulating MAFB expression in ovarian cancer cells. **(A)** Western blot assays were used to assess MAFB expression after MAFB knockdown in RECQL4-overexpressing ovarian cancer cells. **(B)** A growth curve was generated to evaluate the effect of MAFB knockdown on viability changes induced by RECQL4 in HEY and SKOV3 cells. **(C)** A colony formation assay was performed to evaluate the effect of MAFB knockdown on the clone formation induced by RECQL4 in HEY and SKOV3 cells. **(D)** Quantitative analysis of the data in panel **(C)**. **(E)** Transwell assays were performed to evaluate the effect of MAFB knockdown on the invasion induced by RECQL4 in HEY and SKOV3 cells. **(F)** Quantitative analysis of the data in panel **(E)**. *P < 0.05, **P < 0.01.

To confirm that the MAFB acted downstream of RECQL4, rescue experiments were performed. Transiently transfection of MAFB siRNA into HEY and SKOV3 cells which stably overexpressed RECQL4, resulted in repression of MAFB protein expression (Figure 6A). CCK-8 and colony formation assays revealed that silencing MAFB significantly reduced RECQL4-induced cell proliferation (Figures 6B–D). The migration and invasion abilities induced by RECQL4 overexpression were attenuated by MAFB knockdown (Figures 6E,F). Together, these data suggest that the MAFB is involved in RECQL4-mediated oncogenic behavior of ovarian cancer cells.

### RECQL4 Is Negatively Regulated by miR-10a-5p in Ovarian Cancer Cells

**FIGURE 7:**
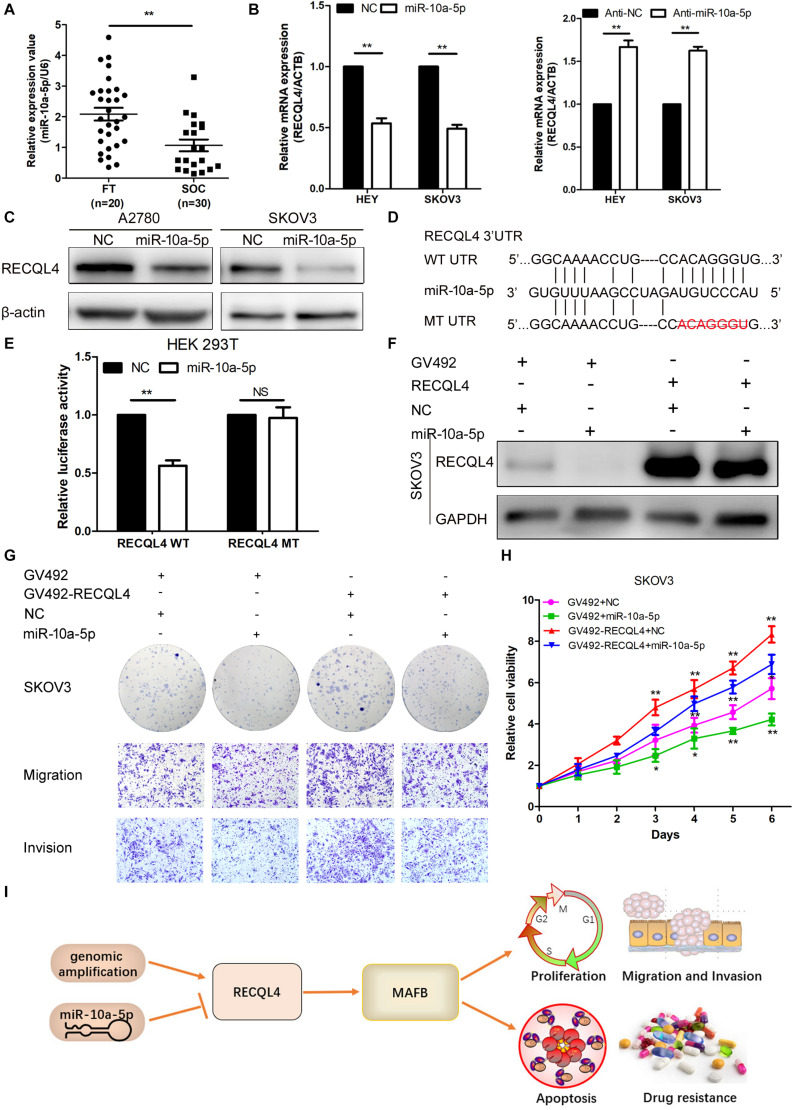
miR-10a-5p suppressed RECQL4 mRNA expression by binding to its 3′UTR. **(A)** Relative expression of miR-10a-5p in 20 FT tissues and 30 ovarian cancer tissues, as determined by qRT-PCR. **(B)** qRT-PCR analysis of RECQL4 mRNA expression after miR-10a-5p knockdown or overexpression in HEY and SKOV3 cells. **(C)** Western blot analysis of RECQL4 protein expression after miR-10a-5p overexpression in ovarian cancer cells. **(D)** Potential binding sequence of miR-10a-5p in the 3′UTR of RECQL4. The wild-type potential binding sequence (ACAGGGU) was changed to the mutant type through deletion of seed sequences. **(E)** Luciferase assays showing that miR-10a-5p mimics decreased the luciferase activity of RECQL4-wild-type (WT) in HEK293T cells, while RECQL4-MT (mutant type) was unaltered. **(F)** Western blotting of RECQL4 expression after miR-10a-5p overexpression in RECQL4-overexpressing and control cells. **(G)** Colony formation assays and transwell assays showed that miR-10a-5p overexpression decreased the cell proliferation, migration, and invasion induced by RECQL4 overexpression. **(H)** CCK-8 assays showed that miR-10a-5p overexpression reduced cell viability induced by RECQL4 overexpression. **(I)** Schematic showing the function of RECQL4 in the proliferation, invasion, and chemoresistance of ovarian cancer. The ovarian cancer tissue samples used in the qRT-PCR and western blot assay are HGSOC. *P < 0.05, **P < 0.01.

To determine the upstream regulator of RECQL4, we used starBase^6^ to predict potential microRNAs binding to RECQL4. Based on the microRNA expression in ovarian cancer tissues, miR-10a-5p expression was found to be downregulated in ovarian cancer compared with fresh-frozen FT tissues, so it was considered a potential functional regulator of RECQL4 (Figure 7A). We next examined whether RECQL4 expression was directly modulated by miR-10a-5p. RECQL4 mRNA and protein expression levels were decreased following transfection with miR-10a-5p mimics, while treatment with an inhibitor increased the expression of RECQL4 in ovarian cancer cells (Figure 7B,C). As shown in Figure 7D, there is a potential conserved binding site in the 3′UTR of RECQL4. To confirm that miR-10a-5p directly modulates RECQL4, dual luciferase reporter assays were performed. We found that miR-10a-5p overexpression reduced the luciferase activity in cells transfected with the wild type 3′UTR of RECQL4 but not in cells with mutant 3′UTR (Figure 7E). This result indicate that RECQL4 is a direct downstream target of miR-10a-5p and RECQL4 overexpression in ovarian cancer may be attributed to the reduced expression of miR-10a-5p.

### miR-10a-5p Inhibits Proliferation and Invasion Through RECQL4 in Ovarian Cancer Cells

To investigate the functionality of miR-10a-5p, we transfected HEY and SKOV3 cells with a miR-10a-5p mimic (miR-10a-5p), inhibitor (anti-miR-10a-5p), and their respective negative controls (NC or anti-NC) for 48 h. The overexpression of miR-10a-5p suppressed proliferation, migration, and invasion of ovarian cancer cells, while the inhibition of miR-10a-5p led to increased viability and mobility (Supplementary Figure S5A–D). To further explore whether miR-10a-5p participates in the RECQL4-mediated enhanced cell proliferation and metastasis, miR-10a-5p mimics or negative controls were transfected into SKOV3 cells stably overexpressing RECQL4, and the protein expression of RECQL4 was assessed by western blotting (Figure 7F). CCK-8 and colony formation assays indicated that the effect of RECQL4-mediated cell proliferation was partially attenuated by treatment with a miR-10a-5p mimic (Figures 7G,H). Transwell assays revealed that the migration and invasion abilities stimulated by RECQL4 overexpression were partially reversed (Figure 7G). These findings suggest that RECQL4 mediates the effects of miR-10a-5p on cell proliferation and invasion in ovarian cancer (Figure 7I).

## Discussion

Several studies have verified that somatic copy number alterations and homologous recombination pathway alterations are a unique feature of HGSOC (20, 21). RECQL4 has been found to be highly amplified in many cancers (22). Consistently, amplification of RECQL4 is related to the development of breast and oral cancer (7, 23, 24). Depletion of RECQL4 decreased homologous recombination and non-homologous end joining efficiency by over 60% in U2OS cells (25). However, the expression, clinical significance, and molecular mechanism of RECQL4 have not been completely characterized. In this study, RECQL4 genomic amplification was found in 27% of sequenced cases and was positively correlated with mRNA expression in a cohort from TCGA. RECQL4 expression was significantly upregulated in ovarian cancer tissues when compared to normal FT tissues. Correlation analysis between RECQL4 and clinical significance found that high expression of RECQL4 was positively associated with serum CA125 level and omental metastasis, and indicated shorter OS. These data highlight that RECQL4 could be a novel prognostic biomarker of ovarian cancer.

Recent studies have demonstrated that DNA damage and repair genes favor cancer cell growth and survival (26, 27). In this study, we demonstrated that RECQL4 overexpression promoted the growth, clone formation, and invasion of ovarian cancer cells In contrast, RECQL4 knockdown reduced cell proliferation and invasion capacity. Meanwhile, flow cytometry analysis showed that RECQL4 silencing increased G1 arrest and decreased the number of cells in S phase to increase the proportion of apoptotic cells. These results indicate that RECQL4 promotes the progression of ovarian cancer by acting as an oncogene.

Preclinical evidence suggests that the restoration of homologous recombination repair may lead to resistance to cisplatin and PARP inhibitor (PARPi) (28–30). RECQL4 has been linked to platinum chemotherapy resistance in gastric cancer, breast cancer and pediatric osteosarcoma (7, 31, 32). In this study, RECQL4 was found to be significantly increased in cisplatin-resistant ovarian cancer cell lines, while knockdown conveyed higher sensitivity to cisplatin. Furthermore it was found the BRCA mutant cell line UWB1.289 was highly sensitive to RECQL4 amplification and silencing, resulting in a significant enhancement and reduction in cell viability, respectively. These results indicate that targeting RECQL4 may be a feasible therapeutic strategy for ovarian cancer patients, including those with BRCA mutations.

High-throughput sequencing was performed to reveal the molecular mechanism of ovarian cancer cells with or without RECQL4 knocked down. MAFB became a point of focus, as its expression was found to be elevated in ovarian cancer in the TCGA cohort. MAFB expression positively correlated with RECQL4 expression, and MAFB expression was increased in RECQL4-overexpressing cells but decreased in RECQL4-knockdown cells. MAFB belongs to the Maf transcription factor family and mediates the differentiation and development of many cells and tissues (33–35). A growing number of studies have shown that MAFB plays a crucial role in tumorigenesis. MAFB was markedly upregulated and promoted HCC growth through upregulation of Cyclin D1 (36). MAFB amplification was identified in colorectal cancer, and SUMOylated MAFB promoted colorectal cancer tumorigenesis by directly regulating CDK6 (37). This study suggested that MAFB silencing inhibited the proliferation and invasion of ovarian cancer cells. Moreover, the proliferation and invasion induced by RECQL4 overexpression were attenuated by MAFB silencing. Therefore, we conclude that the MAFB is a critical effector of RECQL4. Whether RECQL4 directly regulates MAFB will be elucidated in our future studies.

miR-10a-5p is a guide strand of miR-10a that exists in evolutionarily conserved loci within Homeobox gene clusters (38). miR-10a-5p promoted pancreatic ductal adenocarcinoma cells migration and invasion and enhances gemcitabine resistance by directly targeting TFAP2C (39). miR-10a-5p inhibition suppressed cholangiocarcinoma cells proliferation by regulating the PTEN-Akt pathway (40). However, miR-10a-5p expression was significantly decreased, and inhibited the proliferation of breast cancer cells (41). miR-10a-5p was significantly lower in ovarian cancer samples than healthy controls (42). Nevertheless, the function and underlying mechanism of miR-10a-5p action in ovarian cancer remain unclear. In this study, we identified miR-10a-5p as a putative upstream regulator of RECQL4 through bioinformatics analysis. miR-10a-5p was downregulated in ovarian cancer cells, and inhibited proliferation and invasion. Furthermore, miR-10a-5p was shown to suppress RECQL4 by directly binding to its 3′UTR, while RECQL4 overexpression enhanced cell proliferation and motility was partially attenuated by treated with miR-10a-5p mimics. These results highlight that miR-10a-5p serves as a tumor suppressor in ovarian cancer, where it mediates the oncogenic effects of RECQL4.

## Conclusion

In conclusion, our results revealed that RECQL4 amplification and miR-10a-5p suppression resulted in high RECQL4 expression in ovarian cancer. The overexpression of RECQL4 in ovarian cancer patients was associated with poor clinical outcomes. Moreover, upregulated RECQL4 promoted the growth and invasion and reduced olaparib/cisplatin sensitivity of ovarian cancer cells by increasing MAFB expression. Taken together, these data elucidate a novel miR-10a-5p/RECQL4/MAFB axis that regulates the biological functions of ovarian cancer cells. RECQL4 can be used as a potential prognostic marker and a novel drug target for ovarian cancer.

## Data Availability Statement

The raw data supporting the conclusions of this article will be made available by the authors, without undue reservation, to any qualified researcher.

## Ethics Statement

The studies involving human participants were reviewed and approved by the Ethics Committee of Shandong University. The patients/participants provided their written informed consent to participate in this study. The animal study was reviewed and approved by Ethics Committee of Shandong University.

## Author Contributions

BK and YL designed and interpreted this study. LG and YL undertook the data acquisition, analysis, and interpretation. KS, LC, PZ, and HM were responsible for technical support. LG and YL wrote and modified the manuscript. CZ and JP collected the clinical samples. CY, SY, and YF analyzed the clinical prognosis. All authors read and approved the final manuscript.

## Conflict of Interest

The authors declare that the research was conducted in the absence of any commercial or financial relationships that could be construed as a potential conflict of interest.

## Footnotes

1 https://www.cbioportal.org/

2 https://www.cell.com/pb-assets/consortium/pancanceratlas/pancani3/index.html

3 http://ualcan.path.uab.edu/

4 https://portals.broadinstitute.org/ccle/about

5 http://kmplot.com/

6 http://starbase.sysu.edu.cn/starbase2/index.php

## Supplementary Material

FIGURE S1 RECQL4 is overexpressed in ovarian cancer and can predict poor prognosis. **(A)** Copy number variation analysis in HGSOC (upper) and pan-cancer (lower) from TCGA. **(B)** Top 20 genomic amplification genes in TCGA HGSOC and pan-cancer. **(C)** Relative RECQL4 expression between SOC and normal tissues using the data from GSE12470 and GSE26712. **(D)** Correlation analysis between RECQL4 copy number variation and mRNA expression from CCLE. **(E)** Heatmap depicting RECQL4 copy number variation and mRNA expression in ovarian cancer cell lines according to data from CCLE. **(F)** Western blot analysis of RECQL4 protein expression between ovarian cancer cell lines and normal fallopian tube epithelial cells. **(G–I)** High mRNA levels of RECQL4 indicated poor overall survival and progression-free survival (PFS), according to the data from the online analysis website Kaplan–Meier Plotter (http://kmplot.com/). ^∗^*P* < 0.05, ^∗∗^*P* < 0.01.

FIGURE S2 RECQL4 enhances proliferation *in vitro*, and its silencing promotes cisplatin-induced apoptosis. **(A)** Colony formation assays were performed to measure the proliferative ability of ovarian cancer cells after RECQL4 knockdown or overexpression. **(B)** Quantitative analysis of the data in panel **(A)**. **(C)** Apoptosis analysis of ovarian cancer cells was performed with annexin V/PI staining and flow cytometry. SKOV3 cells were transfected with siRNAs for 48 h, which was followed by 4 μg/ml cisplatin treatment for 24 h. **(D)** Quantitative analysis of early and late apoptotic cells by CellQuest Pro of Figure 2F and panel **C**. **(E)** Quantitative analysis of cell cycle-related protein levels after RECQL4 knockdown and overexpression in ovarian cancer cells. **(F)** Quantification of apoptosis-related protein levels after RECQL4 knockdown and overexpression in ovarian cancer cells. The results are shown as the means ± SEM of at least three separate experiments. ^∗^*P* < 0.05, ^∗∗^*P* < 0.01.

FIGURE S3 RECQL4 promotes cell mobility *in vitro*. **(A)** Wound healing assays were performed to evaluate the effect of RECQL4 on the mobility of ovarian cancer cells. **(B)** Quantitative analysis of the wound healing assay data. **(C)** Quantitative analysis of EMT-related protein levels after RECQL4 knockdown and overexpression in ovarian cancer cells. Data are the means ± SEM of three experiments. ^∗^*P* < 0.05, ^∗∗^*P* < 0.01.

FIGURE S4 RECQL4 silencing increases the sensitivity of ovarian cancer cells to cisplatin treatment. **(A)** Western blot analysis of the differential expression of RECQL4 in A2780 and A2780/DDP cells. **(B)** Western blot analysis of RECQL4 protein expression with increasing concentrations of cisplatin for 48 h in ovarian cancer cells. **(C)** A growth curve assay was used to evaluate the viability of cells after RECQL4 knockdown and treatment with increasing concentrations of cisplatin for 48 h (A2780/DDP for 72 h). **(D)** Clonogenic assays were used to evaluate colony formation after RECQL4 knockdown and treated with graded concentrations of cisplatin for 48 h. **(E)** IC50 graph summarizing the data in panel **(D)**. Values are the mean ± SEM from three independent experiments. ^∗^*P* < 0.05, ^∗∗^*P* < 0.01.

FIGURE S5 miR-10a-5p inhibits the proliferation, migration, and invasion of ovarian cancer cells *in vitro*. HEY and SKOV3 cells were transiently transfected with miR-10a-5p mimics (miR-10a-5p) and negative control (NC), miR-10a-5p inhibitor (anti-miR-10a-5p) and negative control (anti-NC) for 48 h. **(A)** a viability assay, which was used to measure the effect of miR-10a-5p on the proliferation of the indicated cells. **(B)** Colony formation assays were applied to evaluate the effect of miR-10a-5p on the colony formation of the indicated cells. **(C)** Transwell assays were performed to evaluate the effect of miR-10a-5p on the migration and invasion of the indicated cells. **(D)** Quantitative analysis of the data in Figure 7G. ^∗^*P* < 0.05, ^∗∗^*P* < 0.01.
