# Supplementary material for: RECQL4, Negatively Regulated by miR-10a-5p, Facilitates Cell Proliferation and Invasion via MAFB in Ovarian Cancer
Source: Front Oncol. 2020 Sep 4;10:524128. doi: 10.3389/fonc.2020.524128 (PMC7500455; doi:10.3389/fonc.2020.524128)
Supplement: FIGURE S1 — RECQL4 is overexpressed in ovarian cancer and can predict poor prognosis. (A) Copy number variation analysis in HGSOC (upper) and pan-cancer (lower) from TCGA. (B) Top 20 genomic amplification genes in TCGA HGSOC and pan-cancer. (C) Relative RECQL4 expression between SOC and normal tissues using the data from GSE12470 and GSE26712. (D) Correlation analysis between RECQL4 copy number variation and mRNA expression from CCLE. (E) Heatmap depicting RECQL4 copy number variation and mRNA expression in ovarian cancer cell lines according to data from CCLE. (F) Western blot analysis of RECQL4 protein expression between ovarian cancer cell lines and normal fallopian tube epithelial cells. (G–I) High mRNA levels of RECQL4 indicated poor overall survival and progression-free survival (PFS), according to the data from the online analysis website Kaplan–Meier Plotter (http://kmplot.com/). ∗P < 0.05, ∗∗P < 0.01. [file Data_Sheet_1.docx]

**Supplementary Figures and tables**


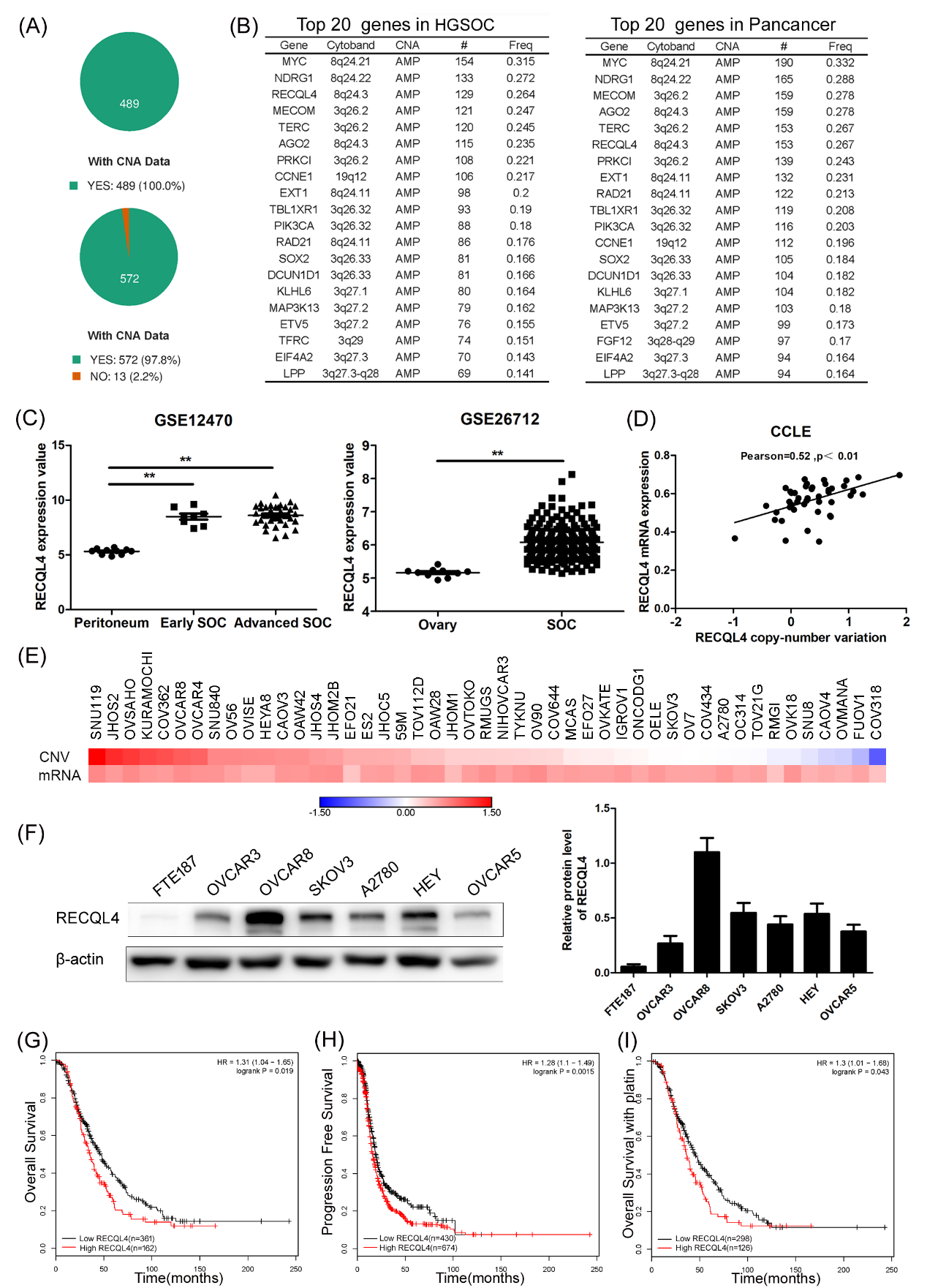


**Supplementary Figure S1. RECQL4 is overexpressed in ovarian cancer and can predict poor prognosis.** **(A)** Copy number variation analysis in HGSOC (upper) and pan-cancer (lower) from TCGA. **(B)** Top 20 genomic amplification genes in TCGA HGSOC and pan-cancer. **(C)** Relative RECQL4 expression between SOC and normal tissues using the data from GSE12470 and GSE26712. **(D)** Correlation analysis between RECQL4 copy number variation and mRNA expression from CCLE. **(E)** Heatmap depicting RECQL4 copy number variation and mRNA expression in ovarian cancer cell lines according to data from CCLE. **(F)** Western blot analysis of RECQL4 protein expression between ovarian cancer cell lines and normal fallopian tube epithelial cells. **(G-I)** High mRNA levels of RECQL4 indicated poor overall survival and progression-free survival (PFS), according to the data from the online analysis website Kaplan-Meier Plotter (http://kmplot.com/). *P<0.05, and **P<0.01.


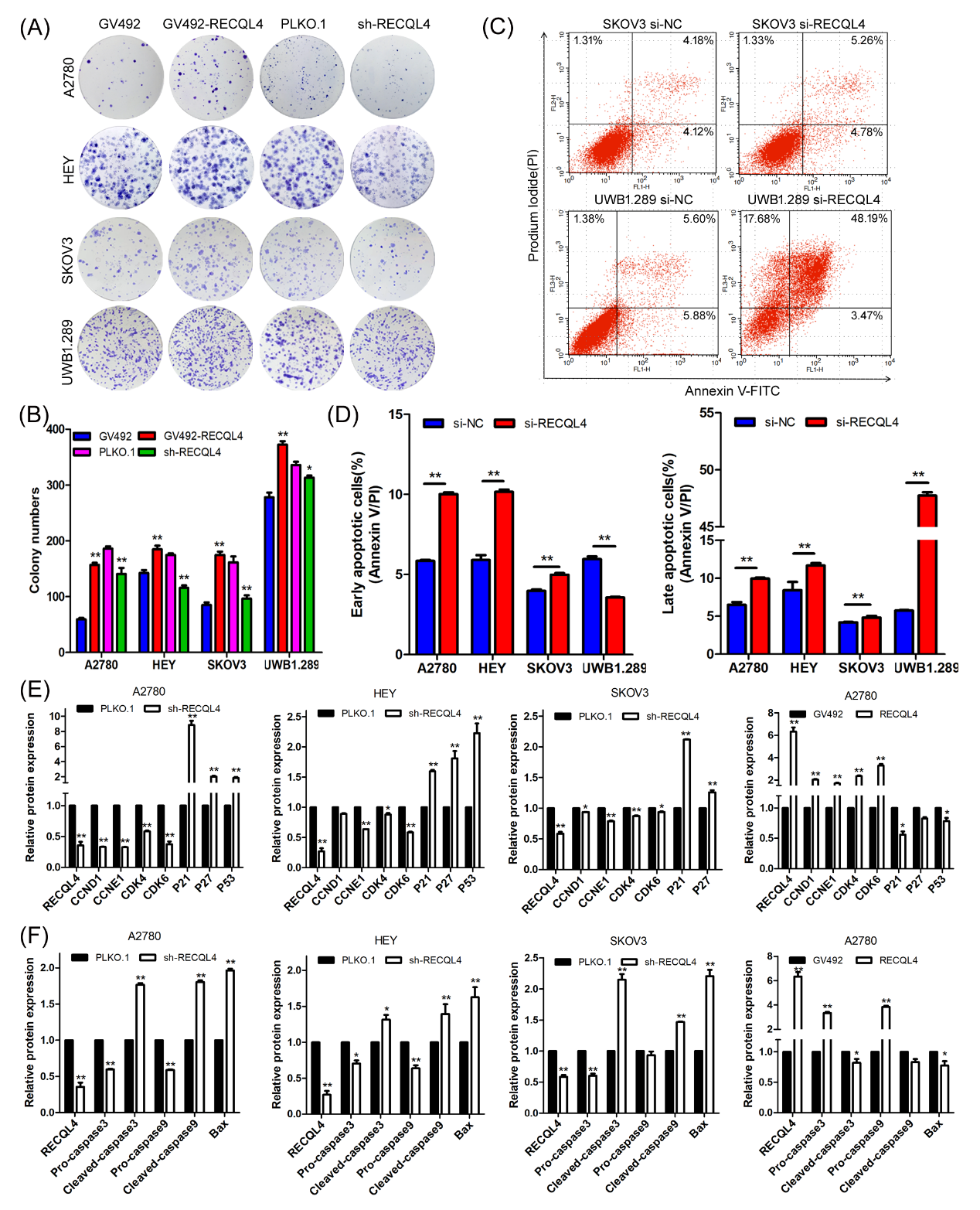


**Supplementary Figure S2. RECQL4 enhances proliferation *in vitro*, and its silencing promotes cisplatin-induced apoptosis. (A)** Colony formation assays were performed to measure the proliferative ability of ovarian cancer cells after RECQL4 knockdown or overexpression. **(B)** Quantitative analysis of the data in Figure A. **(C)** Apoptosis analysis of ovarian cancer cells was performed with annexin V/PI staining and flow cytometry. SKOV3 cells were transfected with siRNAs for 48 hours, which was followed by 4 μg/ml cisplatin treatment for 24 hours. **(D)** Quantitative analysis of early and late apoptotic cells by CellQuest Pro of Figure 2F and Figure S2C. **(E)** Quantitative analysis of cell cycle-related protein levels after RECQL4 knockdown and overexpression in ovarian cancer cells. **(F)** Quantification of apoptosis-related protein levels after RECQL4 knockdown and overexpression in ovarian cancer cells. The results are shown as the means ± SEM of at least three separate experiments. *P<0.05, and **P<0.01.


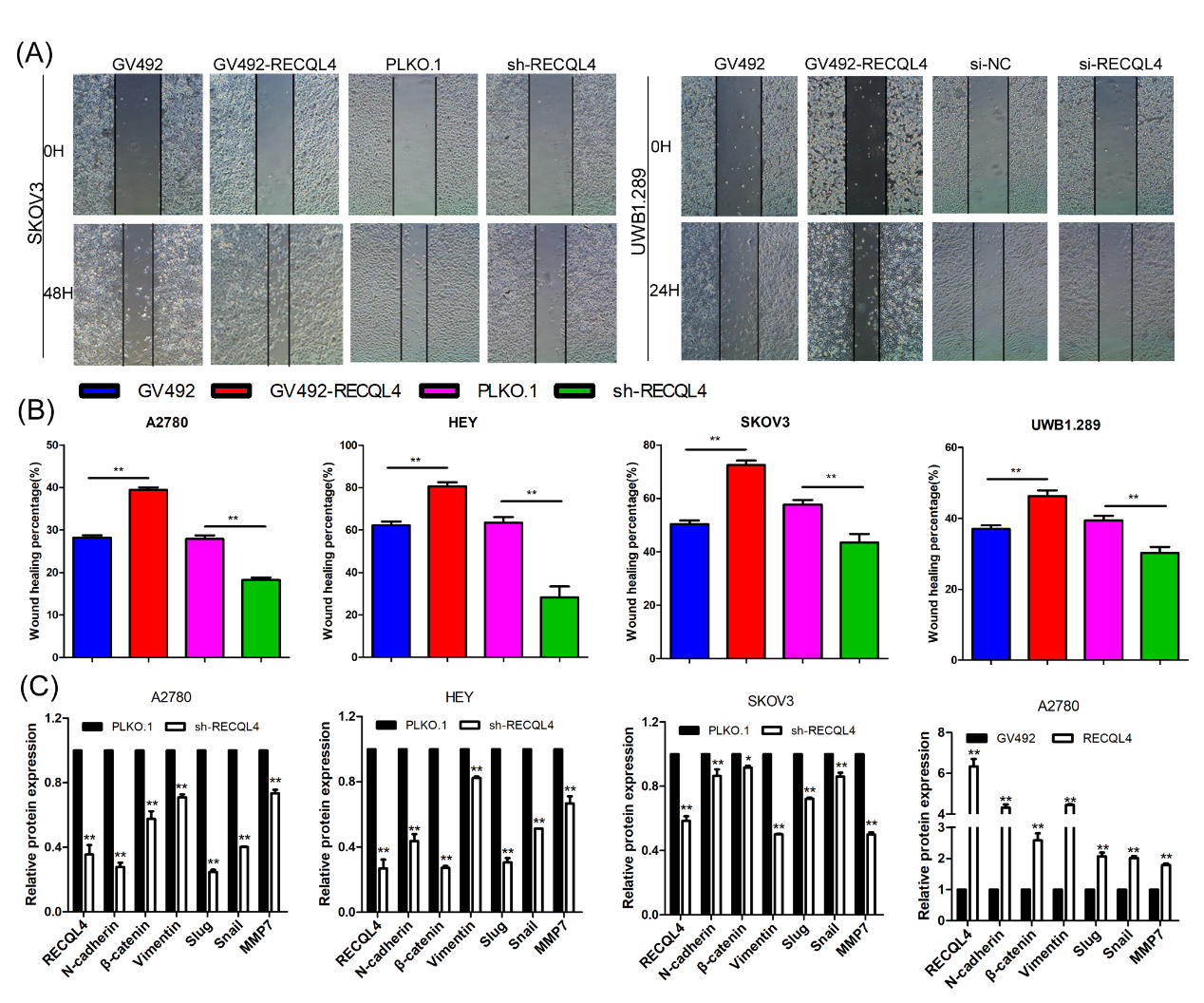


**Supplementary Figure S3. RECQL4 promotes cell mobility *in vitro*. (A)** Wound healing assays were performed to evaluate the effect of RECQL4 on the mobility of ovarian cancer cells. **(B)** Quantitative analysis of the wound healing assay data. **(C)** Quantitative analysis of EMT-related protein levels after RECQL4 knockdown and overexpression in ovarian cancer cells. Data are the means ± SEM of three experiments. *P<0.05, and **P<0.01.


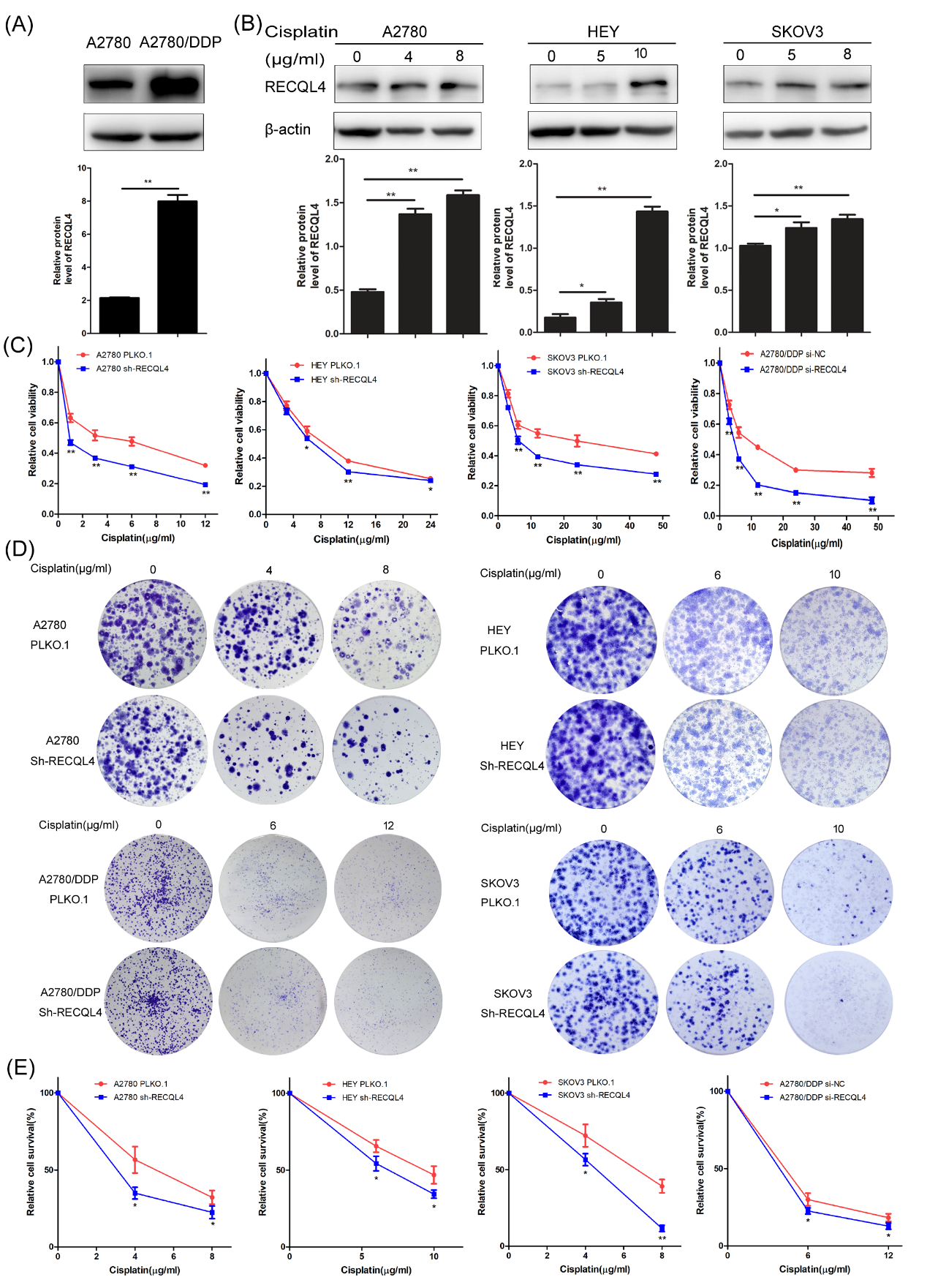


**Supplementary Figure S4. RECQL4 silencing increases the sensitivity of ovarian cancer cells to cisplatin treatment.** **(A)** Western blot analysis of the differential expression of RECQL4 in A2780 and A2780/DDP cells. (B) Western blot analysis of RECQL4 protein expression with increasing concentrations of cisplatin for 48 hours in ovarian cancer cells. **(C)** A growth curve assay was used to evaluate the viability of cells after RECQL4 knockdown and treatment with increasing concentrations of cisplatin for 48 h(A2780/DDP for 72 h). **(D)** Clonogenic assays were used to evaluate colony formation after RECQL4 knockdown and treated with graded concentrations of cisplatin for 48 hours. **(E)** IC50 graph summarizing the data in D. Values are the mean ± SEM from three independent experiments. *P<0.05, and **P<0.01.


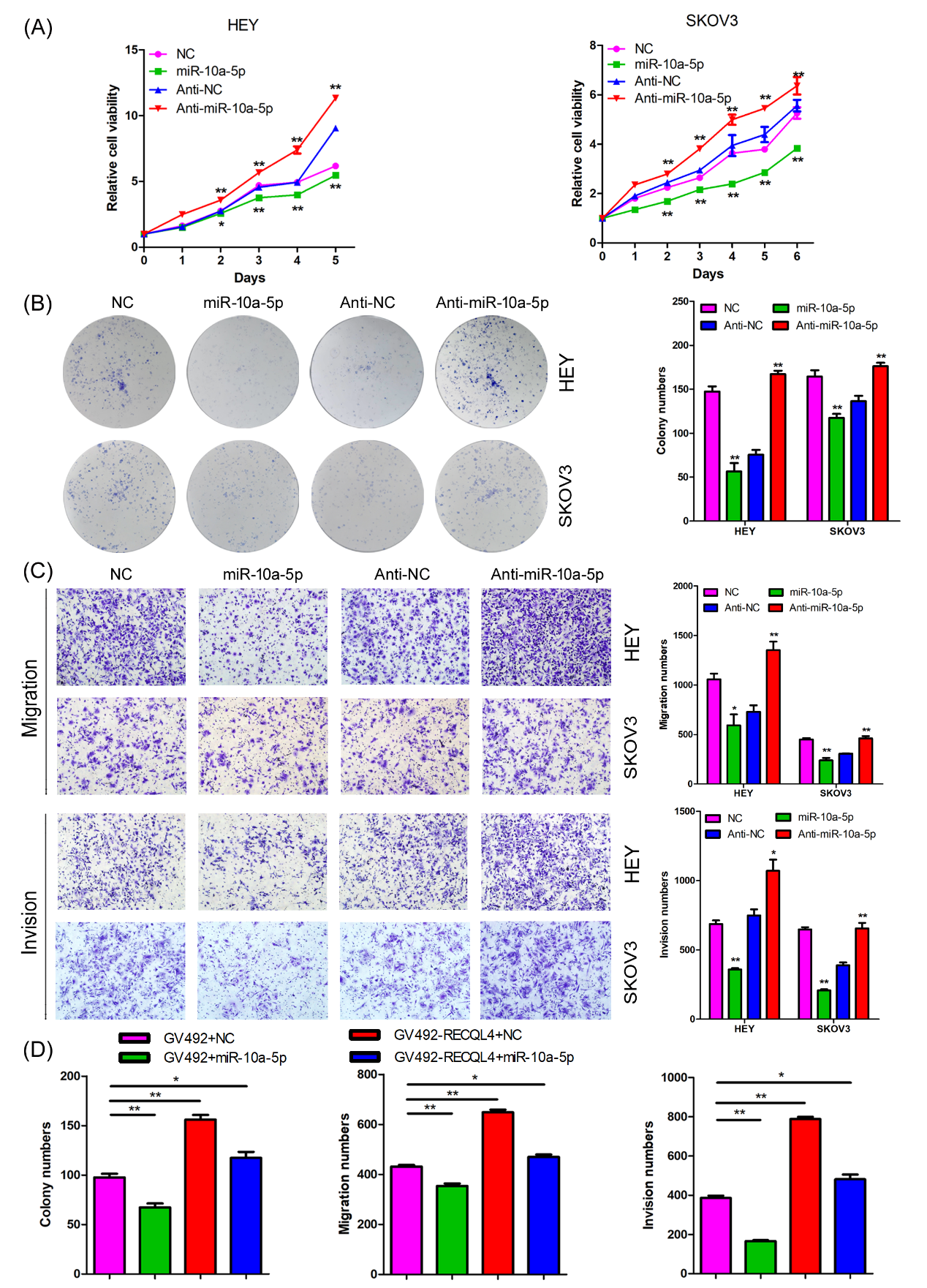


**Supplementary Figure S5. miR-10a-5p inhibits the proliferation, migration, and invasion of ovarian cancer cells *in vitro*.** HEY and SKOV3 cells were transiently transfected with miR-10a-5p mimics (miR-10a-5p) and negative control (NC), miR-10a-5p inhibitor (anti-miR-10a-5p) and negative control (anti-NC) for 48 hours. **(A)** a viability assay, which was used to measure the effect of miR-10a-5p on the proliferation of the indicated cells. **(B)** Colony formation assays were applied to evaluate the effect of miR-10a-5p on the colony formation of the indicated cells. **(C)** Transwell assays were performed to evaluate the effect of miR-10a-5p on the migration and invasion of the indicated cells. **(D)** Quantitative analysis of the data in Figure 7G. *P<0.05, and **P<0.01.

**Supplementary Table S1. Primer and siRNA sequences used in this study.**

| **siRNA sequences** | |
| --- | --- |
|  | **Sequences (5' to 3')** |
| RECQL4-siRNA | CAAUACAGCUUACCGUACATT |
| MAFB-siRNA | CGAGCAACUACCAGCAGAUTT |
| miR-10a-5p mimic | UACCCUGUAGAUCCGAAUUUGUG |
| miR-10a-5p inhibitor | CACAAAUUCGGAUCUACAGGGUA |
| Negative control | UUCUCCGAACGUGUCACGUTT |
| **Primer sequences used for qRT-PCR** | |
| **Gene** | **Primer sequences (5' to 3')** |
| β-actin-F | CATGTACGTTGCTATCCAGGC |
| β-actin-R | CTCCTTAATGTCACGCACGAT |
| RECQL4-F | GATCCTGGCTGGTTACAGCG |
| RECQL4-R | AGTTGTGATTCCTCTGAGCCTA |
| MAFB-F | TCAAGTTCGACGTGAAGAAGG |
| MAFB-R | GTTCATCTGCTGGTAGTTGCT |
| CBLN2-F | AACCGCACCATGACCATCTAT |
| CBLN2-R | GCAAGATCAAAGTGGTTGCCAAT |
| ITGB3-F | CATGAAGGATGATCTGTGGAGC |
| ITGB3-R | AATCCGCAGGTTACTGGTGAG |
| PGPEP1L-F | GAGATGCAGGCAGATACGTCT |
| PGPEP1L -R | CAGCCCTTTCCATGATGCAG |
| GPR78-F | CCACCAGGAAGATTGGCATTG |
| GPR78-R | CTTGCTGTAGGTCAGGCACT |
| MTSS1-F | GAACGTGGCCGATTCTGTACC |
| MTSS1-R | GTGGCGTCTGATACGACCAG |
| AHRR-F | GCGCCTCAGTGTCAGTTACC |
| AHRR-R | GAAGCCCAGATAGTCCACGAT |
| SKAP2-F | TCAGGGGATAAGAGCACTGATT |
| SKAP2-R | ACATCACCACGCTTAAATGACAA |
| IGFBP3-F | AGAGCACAGATACCCAGAACT |
| IGFBP3-R | GGTGATTCAGTGTGTCTTCCATT |
